# Liposomal and Deoxycholate Amphotericin B Formulations: Effectiveness against Biofilm Infections of *Candida* spp.

**DOI:** 10.3390/pathogens6040062

**Published:** 2017-12-01

**Authors:** Célia F. Rodrigues, Mariana Henriques

**Affiliations:** Centre of Biological Engineering (CEB), LIBRO—Laboratório de Investigação em Biofilmes Rosário Oliveira, University of Minho, 4710-057 Braga, Portugal; c.fortunae@gmail.com

**Keywords:** amphotericin B, liposomal, deoxycholate, *Candida* spp., biofilms, drug resistance

## Abstract

Background: candidiasis is the primary fungal infection encountered in patients undergoing prolonged hospitalization, and the fourth leading cause of nosocomial bloodstream infections. One of the most important *Candida* spp. virulence factors is the ability to form biofilms, which are extremely refractory to antimicrobial therapy and very difficult to treat with the traditional antifungal therapies. It is known that the prophylaxis or treatment of a systemic candidiasis are recurrently taken without considering the possibility of a *Candida* spp. biofilm-related infections. Therefore, it is important to assess the effectiveness of the available drugs and which formulations have the best performance in these specific infections. Methods: 24-h-biofilms of four *Candida* spp. and their response to two amphotericin B (AmB) pharmaceutical formulations (liposomal and deoxycholate) were evaluated. Results: generally, *Candida glabrata* was the less susceptible yeast species to both AmBs. MBECs revealed that it is therapeutically more appealing to use AmB-L than AmB-Deox for all *Candida* spp. biofilms, since none of the determined concentrations of AmB-L reached 10% of the maximum daily dose, but both formulations showed a very good capacity in the biomass reduction. Conclusions: the liposomal formulation presents better performance in the eradication of the biofilm cells for all the species in comparison with the deoxycholate formulation.

## 1. Introduction

Infections caused by *Candida* spp. have increased significantly in the past 30 years, becoming a substantial cause of morbidity and mortality. This is particularly critical in immunologically compromised individuals, and in patients submitted to continuous treatment with broad-spectrum antibiotics, to invasive procedures, and with medically implanted devices, which can cause both superficial and systemic infections [1,2]. Although *Candida albicans* is, generally, the most frequently isolated species, there has also been a noteworthy upsurge in the frequency of non-*Candida albicans Candida* (NCAC) species, such as *Candida glabrata*, *Candida parapsilosis* and *Candida tropicalis* [3]. *Candida* spp. pathogenicity is mediated by a number of virulence factors, including the ability to adhere to medical devices and to host cells, which often leads to the formation of biofilms [4]. Biofilms are biological communities with an extraordinary degree of organization, in which microorganisms form structured, coordinated, and functional communities, embedded in a self-created extracellular matrix [1,2,5]. The formation of *Candida* spp. biofilms raises significant clinical issues because of an additional increase in antifungal drug resistance, as well as increased evasion of host immune defences. Furthermore, biofilm development on medical devices can cause the failure of the device and may turn into a source of future infection [6,7,8]. 

Polyenes are among the most effective drugs for the treatment of systemic *Candida* spp. infections, specifically AmB [9,10]. AmB binds to the ergosterol of the fungal cell membrane, establishing transmembrane aggregate pores, causing membrane depolarization with a subsequent increase in membrane permeability to monovalent protons and cations. This allows the passage of intracellular molecules to the external environment, initiating an osmotic imbalance and, finally, cell death [11,12,13]. This drug, produced by *Streptomyces nodosus*, is part of the macrolides class, and is characterized by a macrocyclic ring lactone, with a hydrophobic and one hydrophilic domain, giving it an amphipathic characteristic that confers low solubility in aqueous solutions at physiological pH. With low bioavailability via oral administration, AmB is a highly hydrophobic weak base, with a low aqueous solubility of approximately 1 ng/mL at pH 7 [14,15], needing to beproduced as a complex with another agent to enable its clinical use, assodium deoxycholate. The possible administration routes are intravenous, intra-articular, intravesical, intrathecal, in injuries and applied in surgical sites [12,13,16].

AmB deoxycholate (AmB-Deox) has been used during recent years due to the rise in the number of immunosuppressed patients suffering invasive fungal infections, but has been related to a high rate of side effects, particularly renal toxicity [13,17]. Therefore, other formulations have been developed: a lipid formulation (liposomal, AmBisome^®^, Gilead Sciences, Foster City, CA, USA), a lipid complex (Abelcet^®^, Sigma Tau Pharmaceuticals, Pomezia, Italy), and a colloidal suspension (Amphocil^®^, Penn Pharmaceuticals, Ltd., Tredegar, UK), which share the same antifungal spectrum but differ in efficacy and toxicity [13,17]. The liposomal formulation (AmB-L) is constituted by 50–100 µm spheres, and is composed of hydrogenated phosphatidylcholine soy, 25% cholesterol, sterically attached to distearylphosphatidyl glycerol (DSPG) and AmB. Every AmB molecule within the liposome is complexed with DSPG and cholesterol, which allows it to escape the initial clearance of the sarcoplasmatic reticulum; but once captured, the concentrations in the liver and spleen increase as it decreases in the plasma. Consequently, it is guaranteed that there are no AmB-L residues in blood, reaching the highest serum concentrations [13,18,19,20,21,22].

The first cases of resistance to polyenes’ treatment are related with the increase of systemic infections, many of them with primary or intrinsic resistance to AmB and consistently associated with high mortality rate [23,24,25]. These cases have been increasing, but fortunately the studies still recognize the high effectiveness of AmB-Deox and AmB-L on planktonic cells or in the prevention of the biofilm formation of *Candida* spp. [26,27,28,29,30,31]. On the other hand, fewer researchers have performed studies specifically on the activity of the two formulations on matured biofilms [32,33]. Since these communities are known to be responsible for the most aggressive systemic infections [2], the aim of this study was to evaluate the efficacy of the AmB liposomal formulation (AmbiSome^®^) compared to the original, deoxycholate (Fungizone^®^) in eliminating the cells derived from matured biofilms of the four most common *Candida* spp. found in hospitals: *Candida albicans*, *Candida glabrata*, *Candida parapsilosis* and *Candida tropicalis*.

## 2. Results and Discussion

The prevalence of invasive fungal infections will unquestionably continue to grow due to the constant use of immunosuppressive and broadspectrum antimycotic therapy, the increase in the number of patients at risk in medical care and particularly with the rise in the numbers of severely immunocompromised patients [1,34,35,36]. Currently, echinocandins are considered the first-line antifungals for treating systemic candidiasis [37,38]. Possibly as a result of this, both breakthrough infections and acquired resistance mutations in certain *Candida* spp. have been reported (especially for *C. glabrata*), which makes the management of invasive candidiasis a permanent challenge [39,40]. It is therefore vital to perform a constant assessment of whether antifungal drugs are still effective, appropriate, and clinically safe. AmB, a polyene antifungal drug, binds to ergosterol, and also induces the accumulation of reactive oxygen species, resulting in multiple deleterious and fungicidal effects on fungal cells, which probably explains the low rate of resistance events associated with this drug [26,41]. Still, AmB also has the ability to bind to cholesterol, although with lower affinity, which is assumed to be connected to its greater toxic potential. Due to the high frequency of nephrotoxicity using AmB-Deox [42], the pharmaceutical industry has produced formulations with lipids, namely AmB-L [13,43,44,45]. This work focused on *Candida* spp. infections resulting exclusively from biofilm cells—which are recognized as having a great impact in nosocomial infections, although they are still poorly understood [46]—and how they responded to two different formulations of AmB.

The results of MIC and MFC can be observed in Table 1. All four *Candida* spp. were considered susceptible to both AmB formulations, according to the most recent EUCAST breakpoints [47]. Generally, AmB-Deox had a better performance than AmB-L in both tests, showing elimination of spectrophotometric growth (MIC) or elimination, at least, of 2 Log_10_ CFU/cm^2^ of the initial inoculum (MFC) at lower concentrations. With regard to the MIC results, *C. tropicalis* ATCC750 was the most tolerant to AmB-Deox (0.5 mg/L, compared to the rest, 0.25 mg/L) and *C. albicans* SC5314 the most sensitive to AmB-L (0.5 mg/L, compared to the rest, 1 mg/L). AmB-Deox is a colloidal dispersion with a size of 0.035 nm, in contrast to the ~0.080 nm of size of the spherical AmB-L [13,48]. This fact probably influenced the immediate cellular penetration in the free planktonic cells, enhancing it in the case of the AmB-Deox, when comparing both formulations. In particular, for two of the studied species (*C. glabrata* ATCC2001 and *C. parapsilosis* ATCC22019) AmB-L required a concentration 4 times higher (1 mg/L) than that required when using AmB-Deox (0.25 mg/L). Similar results have been shown by other authors regarding *C. glabrata* species [49,50,51,52,53]. *C. albicans* SC5314 was the species that demanded the lowest drug concentrations, for both formulations, responding very well to this polyene, as it has been indicated before [49,54,55,56,57,58]. With regard to the MFC results (Table 1), the differences between the four species were not so evident. Generally, the concentration that points to the inhibition of growth (MIC) is closer to cell death (MFC) for AmB-L than for AmB-Deox. Using AmB-L, the concentration used for NCAC species was 1.5 times higher than for MIC; and for *C. albicans* SC5314, the value was 3 times higher, showing a higher drug tolerance in this species, which has been demonstrated previously [59]. With AmB-Deox, these variations were more noteworthy. In fact, it seems to have an advantage in the use of AmB-L over AmB-Deox. These variations on *Candida* spp. responses between AmB-Deox and AmB-L can determine the outcome in an infection treatment, especially in biofilm infections, since it is known that the inhibition of growth does not always lead to cell death, but sometimes to cell dormancy, or even the appearance of tolerant or persister cells [59,60,61,62,63,64,65,66]. None of the drug formulations demanded high *in vitro* doses to show efficacy; thus, they were shown to be good options for the treatment of planktonic *Candida* spp. infections.

**Figure 1:**
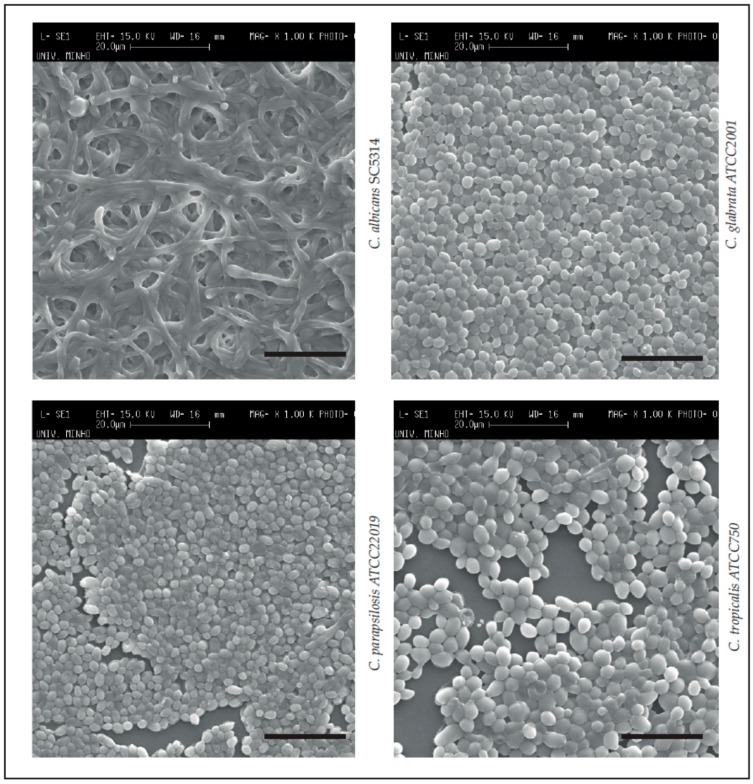
SEM images of matured biofilms of *Candida albicans* SC5314, *Candida glabrata* ATCC2001, *Candida parapsilosis* ATCC22019 and *Candida tropicalis* ATCC750. Magnification: 1000× (Measure bar = 20 µm).

Next, in order to visualize the appearance of the matured biofilms of the four *Candida* spp., SEM images were obtained (Figure 1). It was confirmed that all *Candida* spp. were able to form structured biofilms under the culture conditions applied. Specifically, *C. albicans* SC5314 demonstrated a biofilm with high hyphae quantity and entanglement [2,67]. This morphological change (from yeast to hyphae) can influence biofilm formation and stability [2,68]. *C. glabrata* ATCC2001 formed biofilms constituted by yeasts in a long continuous carpet [2,69,70] and *C. parapsilosis* ATCC22019 a continuous biofilm carpet with clumped blastospores [2,71]. Finally, the *C. tropicalis* ATCC750 biofilm could be described as chains of cells with high amounts of extracellular material [2,72,73]. These strong biofilms have been related to higher pathogenicity, virulence and resistance, and to difficulties in drug diffusion [2,74,75,76,77].

With regard to the main goal of this study—the treatment and eradication of the biofilm (MBEC, Table 2)—the outcomes changed significantly. Both AmB-L and AmB-Deox required a drug concentration between 4 and 8 times greater to eliminate the biofilm cells compared to the corresponding planktonic cells. Since AmB-Deox and AmB-L have very different permitted daily doses for a systemic candidiasis (AmB-Deox: 1.2 mg/kg/day for an adult of 70 kg, and AmB-L: 6 mg/kg/day [78,79]), in order to ease the comparison of the results, the data were transformed into percentage of maximum permitted daily dose (Table 2). 

Generally, AmB-L showed a better response on the 24-h-pre-formed biofilms. Excepting *C. glabrata* ATCC2001, the three other *Candida* spp. needed 2 mg/L of AmB-Deox to eliminate the biofilm cells, which represents approximately 12% of the maximum of the daily dose for this formulation. *C. glabrata* ATCC2001 presented a resistant pattern, requiring 4 mg/L (meaning almost 24% of the daily dose), which was not a total surprise, since for this species, this performance, as well as, resistance cases with polyenes, have already been reported [30,80,81,82,83]. With AmB-L, the values were slightly more variable. *C. parapsilosis* ATCC22019 was the least resistant species [47], with 2 mg/L (2.38% of the maximum dose), followed by *C. albicans* SC5314, with 3 mg/L (3.57% of the maximum dose) (Table 2). The MBEC values obtained were higher than previously reported for other *C. albicans* strains [84,85,86], but were similar for *C. parapsilosis* [87,88]. The differences observed could be due to alterations in the biofilm formation conditions, and the fact that the MBEC evaluations methods used were not identical. Nonetheless, Prazynka and colleagues [30] had parallel outcomes in 24-h-pre-formed biofilms. *C. glabrata* ATCC2001 and *C. tropicalis* ATCC750 presented a clearer biofilm resistance profile, with results of ≥8 mg/L and ≥9.52% of the maximum dose (Table 2). Comparable concentrations for these two species have already been reported by other authors [89,90,91,92,93]. It is noted, though, that when comparing the percentages of clinical doses, the required concentrations to eliminate the biofilm are therapeutically more appealing when using AmB-L than AmB-Deox for all *Candida* spp., since none of the determined concentrations of AmB-L even reached 10% of the maximum daily dose (Table 2). The differences between the percentages of the two AmB formulations were statistically significant for each species (*p* < 0.001).

Finally, with regard to biofilm biomass reduction (Table 3), it was possible to observe a dependence on species and AmB formulation; but generally, both AmBs were shown to have good performance, with reductions between 34.64% and 89.58% for AmB-Deox, and between 43.78% and 70.72% for AmB-L. *C. tropicalis* ATCC750 and *C. albicans* SC5314 showed a pronounced biofilm reduction, with only 0.25 mg/L of AmB-Deox (~90% and ~70%, respectively), but the MBEC values demonstrated that the same biofilm cells required 8 times more drug concentration (2 mg/L) to be eliminated. The opposite happened with *C. glabrata* ATCC2001 and *C. parapsilosis* ATCC22019. These two species demanded a concentration near to the MBEC value to eliminate 50% of the biofilm, showing the capacity to produce robust biofilms on abiotic surfaces, as has been previously described [94]. Regarding AmB-L, *C. albicans* SC5314 was the species that required the lowest AmB-L concentration to eliminate the highest percentage of its biomass (70.72%). In contrast, *C. glabrata* ATCC2001 needed a higher dose, confirming the biofilm’s strong structure [95]. These findings have also been reported by other authors [90,96].

It is important to note that the obtained results regarding the biomass reductions do not match the susceptibility determinations (planktonics and biofilms), since high biomass reductions were obtained with lower (than MBEC) AmB concentrations for both formulations. It is known that, in situations of drug stress, some species/strains block the production and exportation of certain biofilm matrices’ compounds (e.g., proteins in *C. glabrata*) [97] and that, in these cases, subpopulation cells with increased tolerance to AmB or even persister cells can arise, exclusively in the biofilms [61,63,98]. Although these facts are acknowledged, it is still widely admitted that the biofilm drug resistance in *Candida* spp. remains to be totally explained, and is most likely multifactorial in nature. 

## 3. Conclusions

In conclusion, compared with conventional amphotericin B, the liposomal formulation offers a better safety profile in both adults and children, and accumulates in tissue, which is therapeutically advantageous. Our results now show that AmB-L is a good option for the treatment of infections directly associated with *Candida* spp. biofilm cells. Continuous clinical observations are essential to measuring the activity of AmB-L against yeasts, in order to detect strains with low drug susceptibility, thus supporting the most adequate choice of prompt antifungal treatment towards an improved prognosis for the patient. 

## 4. Material and Methods

### 4.1. Organisms and Growth Conditions

Four reference species of *Candida* spp., were used in this study: *C. albicans* SC5314, and three from the American Type Culture Collection (ATCC), *C. glabrata* ATCC2001, *C. parapsilosis* ATCC22019 and *C. tropicalis* ATCC750. For each experiment, yeasts were subcultured on Sabouraud dextrose agar (SDA) (Merck, Darmstadt, Germany) for 24 h at 37 °C. Cells were then inoculated in Sabouraud dextrose broth (SDB) (Merck, Darmstadt, Germany) and incubated for 18 h at 37 °C under agitation at 120 rpm. After incubation, the cells were harvested by centrifugation at 3000× *g* for 10 min at 4 °C and washed twice with phosphate-buffered saline (PBS, pH = 7.5). Pellets were then suspended in RPMI 1640 (Sigma-Aldrich, St. Louis, MO, USA) and the cellular density was adjusted to 1 × 10^5^ cells/mL, using a Neubauer counting chamber.

### 4.2. Antifungal Drugs

AmB-Deox was purchased from Sigma^®^ (Sigma-Aldrich, St. Louis, MO, USA) and AmB-L was supplied by Gilead^®^ (Foster City, CA, USA). Aliquots of 2000 mg/L were prepared using dimethyl-sulfoxide (DMSO) for AmB-Deox and of 1000 mg/L for AmB-L according to the indications of the manufacturer. 

### 4.3. Antifungal Susceptibility Tests

The antifungal susceptibility tests, for both formulations of AmB, were determined using the microdilution method, in accordance with the European Committee on Antimicrobial Susceptibility Testing guidelines [47].

### 4.4. Planktonic Susceptibility Evaluation

#### 4.4.1. Minimum Inhibitory Concentrations (MICs)

The AmB MIC is the lowest concentration, recorded in mg/L, of the drug that inhibits the growth of the yeasts to a predefined degree (e.g., 90% in the case of polyenes) [99]. The MIC provides information on the susceptibility or resistance of the *Candida* spp. to the AmB formulations [47,100].

The AmB concentrations tested were prepared in RPMI 1640 (pH = 7) (Sigma-Aldrich, St. Louis, MO, USA). The inoculum was prepared by suspending five distinct colonies, ≥1 mm diameter from 24 h cultures, in at least 3 mL of sterile distilled water. Then, the inoculum was suspended by vigorous shaking on a vortex mixer for 15 s and the cell density was adjusted to the density of a 0.5 McFarland standard, adding sterile distilled water as required, giving a yeast suspension of 1–5 × 10^6^ colony-forming units (CFUs) CFU/mL. A working suspension was prepared by a dilution of the standardised suspension in sterile distilled water to yield 1–5 × 10^5^ CFU/mL. The 96-well plate was prepared with 100 μL of cell suspension and 100 μL of antifungal agent (0.25, 0.5, 1 and 1.5 mg/L, 2× concentrated) and incubated at 37 °C, during 18–48 h. Controls without antifungal agents were also performed. Finally, the results were visualized by spectrophotometry at 530 nm.

#### 4.4.2. Minimum Fungicidal Concentration (MFC)

The AmB MFC is the lowest concentration, recorded in mg/L, of the drug that reduces the planktonic population to at least 2 Log_10_ CFU per cm^2^. For that determination, in addition to the previous step, 20 µL of each cell suspension treated with AmB-Deox and AmB-L was recovered and placed in a new well, and serial decimal dilutions in phosphate-buffered saline (PBS 0.1 M pH 7.5) were plated onto SDA. Agar plates were incubated for 24 h at 37 °C, and the total number of CFUs was determined. The results were calculated by Log_10_ CFU per cm^2^ (Log_10_ CFUs/cm^2^) and presented by mg/L [101]. 

### 4.5. Biofilm Structure, Susceptibility Evaluation and Biomass Reduction Analysis

#### 4.5.1. Scanning Electronic Microscopy (Biofilm Structure Visualization)

In order to examine the structure of the biofilms of the *Candida* spp., they were observed by scanning electron microscopy. For this, the biofilms formed as described above were dehydrated with ethanol (using 70% ethanol for 10 min, 95% ethanol for 10 min and 100% ethanol for 20 min) and air-dried for 20 min. Samples were kept in a desiccator until the base of the wells was removed for analysis. Prior to observation, the bases of the wells were mounted onto aluminum stubs, sputter-coated with gold, and observed with an S-360 scanning electron microscope (Leo, Cambridge, MA, USA).

#### 4.5.2. Minimum Biofilm Eradication Concentration (MBEC)

The AmB MBEC is the lowest concentration, recorded in mg/L, of the drug able to reduce the biofilm cell population to at least 2 Log_10_ CFU per cm^2^. For this determination, standardized cell suspensions (200 μL) were placed into selected wells of 96-well polystyrene microtiter plates (Orange Scientific, Braine-l’Alleud, Belgium). RPMI 1640 (Sigma-Aldrich, St. Louis, MO, USA) was used without cells, but with antifungal agent, as a negative control. As positive control, cell suspensions were tested without antifungal agent. At 24 h, 100 μL of RPMI 1640 (Sigma-Aldrich, St. Louis, MO, USA) was removed, and an equal volume of fresh RPMI 1640 plus the respective antifungal concentration was added (2, 3, 4, 8 mg/L, 2× concentrated). The plates were incubated at 37 °C for another 24 h, a total of 48 h at 120 rpm. The number of cultivable cells on biofilms was determined by the enumeration of CFUs. For this, after the period of biofilm formation, all medium was aspired, and the biofilms were washed once with 200 μL of PBS to remove non-adherent cells. Then, biofilms were scraped from the wells, and the suspensions were vigorously vortexed for 2 min to disaggregate the cells from the matrix. Serial decimal dilutions in PBS were plated on SDA and incubated for 24 h at 37 °C. The results were calculated as a total of CFUs per unit area (Log_10_ CFUs/cm^2^), and presented by mg/L [101].

#### 4.5.3. Biofilm Total Biomass Quantification—Crystal Violet Staining

Total biofilm biomass was quantified by crystal violet (CV) staining [102]. After biofilm formation, the medium was aspirated, and non-adherent cells removed by washing the biofilms with sterile ultra-pure water. Then, biofilms were fixed with 200 μL methanol, which was removed after 15 min of contact. The microtiter plates were allowed to dry at room temperature, and 200 μL of CV (1% *v*/*v*) were added to each well and incubated for 5 min. The wells were then gently washed twice with sterile, ultra-pure water and 200 μL of acetic acid (33% *v*/*v*) were added to release and dissolve the stain. The absorbance of the obtained solution was read in triplicate in a microtiter plate reader (Bio-Tek Synergy HT, Izasa, Lisbon, Portugal) at 570 nm. Three negatives were performed using sterile ultra-pure water. The results were presented as percentage of reduction of biomass. 

### 4.6. Statistical Analysis 

The assays were performed in triplicate, and on three separate occasions. Results were compared using two-way ANOVA, and Bonferroni’s post-test, using GraphPad Prism 5 software. All tests were performed with a confidence level of 95%.

## Figures and Tables

**Table 1:** Results in MIC and MFC concentrations for both AmB formulations.

Species	MIC (mg/L)		MFC (mg/L)	
	AmB-Deox	AmB-L	AmB-Deox	AmB-L
*Candida albicans* SC5314	0.25	0.5	1	1.5
*Candida glabrata* ATCC2001	0.25	1	1	1.5
*Candida parapsilosis* ATCC22019	0.25	1	1	1.5
*Candida tropicalis* ATCC750	0.5	1	1	1.5

**Table 2:** MBEC values of AmB-Deox and AmB-L and its percentage on the maximum permitted dose used ^‡^.

Species	MBEC (mg/L)			
	AmB-Deox	% of Maximum Permitted Dose ^#^	AmB-L	% of Maximum Permitted Dose ^#^
*Candida albicans* SC5314	2	11.90	3	3.57 ***
*Candida glabrata* ATCC2001	4	23.81	≥8	≥9.52 ***
*Candida parapsilosis* ATCC22019	2	11.90	2	2.38 ***
*Candida tropicalis* ATCC750	2	11.90	≥8	≥9.52 ***

^‡^ The difference between the percentages of both AmB formulations are all statistically significant; *** *p* < 0.001; ^#^ Considered to be the maximum dose allowed for invasive candidiasis [78,79]: AmB-Deox—(0.6–1.2) mg/kg for an adult of 70 kg; AmB-L—(3–6) mg/kg for an adult of 70 kg.

**Table 3:** Percentage of biofilm reduction closer to 50 when using AmB-Deox and AmB-L for reference species of *C. albicans*, *C. glabrata*, *C. parapsilosis* and *C. tropicalis*.

Species	[Drug] mg/L for Biofilm Reduction Closer to 50%			
	[AmB-Deox]	% Max Biofilm Reduction	[AmB-L]	% Max Biofilm Reduction
*Candida albicans* SC5314	0.25	68.56	0.5	70.72
*Candida glabrata* ATCC2001	1.5	51.56	1.5	48.86
*Candida parapsilosis* ATCC22019	1.5	34.64	1	43.78
*Candida tropicalis* ATCC750	0.25	89.58	1	50.66

## References

[1] Rodrigues C., Rodrigues M., Silva S., Henriques M. (2017). *Candida glabrata* Biofilms: How Far Have We Come?. J. Fungi.

[2] Silva S., Rodrigues C., Araújo D., Rodrigues M., Henriques M. (2017). Candida Species Biofilms’ Antifungal Resistance. J. Fungi.

[3] Lass-Flörl C. (2009). The changing face of epidemiology of invasive fungal disease in Europe. Mycoses.

[4] Tomičić Z., Zupan J., Matos T., Raspor P. (2016). Probiotic yeast *Saccharomyces boulardii* (nom. nud.) modulates adhesive properties of *Candida glabrata*. Med. Mycol..

[5] Shields R.K., Nguyen M.H., Press E.G., Kwa A.L., Cheng S., Du C., Clancy C.J. (2012). The presence of an FKS mutation rather than MIC is an independent risk factor for failure of echinocandin therapy among patients with invasive candidiasis due to *Candida glabrata*. Antimicrob. Agents Chemother..

[6] Douglas L.J. (2003). Candida biofilms and their role in infection. Trends Microbiol..

[7] Ramage G., Rajendran R., Sherry L., Williams C. (2012). Fungal biofilm resistance. Int. J. Microbiol..

[8] Zhang L., She X., Merenstein D., Wang C., Hamilton P., Blackmon A., Hu H., Calderone R., Li D. (2014). Fluconazole Resistance Patterns in Candida Species that Colonize Women with HIV Infection. Curr. Ther. Res. Clin. Exp..

[9] Pierce C.G., Srinivasan A., Uppuluri P., Ramasubramanian A.K., López-Ribot J.L. (2013). Antifungal therapy with an emphasis on biofilms. Curr. Opin. Pharmacol..

[10] Delattin N., Cammue B.P.A., Thevissen K. (2014). Reactive oxygen species-inducing antifungal agents and their activity against fungal biofilms. Future Med. Chem..

[11] Lemke A., Kiderlen A.F., Kayser O. (2005). Amphotericin B. Appl. Microbiol. Biotechnol..

[12] Baginski M., Czub J. (2009). Amphotericin B and its new derivatives-mode of action. Curr. Drug Metab..

[13] Botero M.C., Puentes-Herrera M., Cortés J.A. (2014). Lipid formulations of amphotericin. Rev. Chil. Infectol..

[14] Stephens N., Rawlings B., Caffrey P. (2012). Streptomyces nodosus host strains optimized for polyene glycosylation engineering. Biosci. Biotechnol. Biochem..

[15] Storm G., van Etten E. (1997). Biopharmaceutical aspects of lipid formulations of amphotericin B. Eur. J. Clin. Microbiol. Infect. Dis..

[16] Laniado-Laborin R., Cabrales-Vargas M. (2009). Amphotericin B: Side effects and toxicity. Rev. Iberoam. Micol..

[17] Azanza P., Ramon J., Jose B. (2012). Anfotericina B forma liposomal: Un perfil farmacocinético exclusivo. Una historia incabada. Rev. Esp. Quimioter..

[18] Kshirsagar N., Pandya S., Kirodian G., Sanath S. (2005). Liposomal drug delivery system from laboratory to clinic. J. Postgrad. Med..

[19] Adler-Moore J., Proffitt R. (2008). Amphotericin B lipid preparations: What are the differences?. Clin. Microbiol. Infect..

[20] Hamill R. (2013). Amphotericin B formulations: A comparative review of efficacy and toxicity. Drugs.

[21] Patel R. (2000). Amphotericin B colloidal dispersion. Expert Opin. Pharmacother..

[22] Antoniadou A., Dupont B. (2005). Lipid formulations of amphotericin B: Where are we today?. J. Mycol. Med..

[23] Berenguer J., Rodriguez-Tudela J., Richard C., Alvarez M., Sanz M., Gaztelurrutia L., Ayats J., Martinez-Suarez J., *Scedosporium prolificans* Spanish Study Group (1997). Deep infections caused by *Scedosporium prolificans*. A report on 16 cases in Spain and a review of the literature. Medicine.

[24] Boutati E., Anaissie E. (1997). Fusarium, a significant emerging pathogen in patients with hematologic malignancy: Ten years’ experience at a cancer center and implications for management. Blood.

[25] Tritz D., Woods G. (1993). Fatal disseminated infection with Aspergillus terreus in immunocompromised hosts. Clin. Infect. Dis..

[26] Montagna M.T., Lovero G., Coretti C., De Giglio O., Martinelli D., Bedini A., Delia M., Rosato A., Codeluppi M., Caggiano G. (2014). In vitro activities of amphotericin B deoxycholate and liposomal amphotericin B against 604 clinical yeast isolates. J. Med. Microbiol..

[27] Moen M.D., Lyseng-Williamson K.A., Scott L.J. (2009). Liposomal amphotericin B: A review of its use as empirical therapy in febrile neutropenia and in the treatment of invasive fungal infections. Drugs.

[28] Faria-Ramos I., Neves-Maia J., Ricardo E., Santos-Antunes J., Silva A.T., Costa-de-Oliveira S., Cantón E., Rodrigues A.G., Pina-Vaz C. (2014). Species distribution and in vitro antifungal susceptibility profiles of yeast isolates from invasive infections during a Portuguese multicenter survey. Eur. J. Clin. Microbiol. Infect. Dis..

[29] Krogh-Madsen M., Arendrup M.C., Heslet L., Knudsen J.D. (2006). Amphotericin B and Caspofungin Resistance in *Candida glabrata* Isolates Recovered from a Critically Ill Patient. Clin. Infect. Dis..

[30] Prażyńska M., Gospodarek E. (2014). In vitro effect of amphotericin B on *Candida albicans*, *Candida glabrata* and *Candida parapsilosis* biofilm formation. Mycopathologia.

[31] Kiraz N., Oz Y. (2011). Species distribution and in vitro antifungal susceptibility of clinical Candida isolates from a university hospital in Turkey over a 5-year period. Med. Mycol..

[32] Kawai A., Yamagishi Y., Mikamo H. (2015). In vitro efficacy of liposomal amphotericin B, micafungin and fluconazole against non-albicans Candida species biofilms. J. Infect. Chemother..

[33] Da Silva B.V., Silva L.B., de Oliveira D.B.C., da Silva P.R., Ferreira-Paim K., Andrade-Silva L.E., Silva-Vergara M.L., Andrade A.A. (2015). Species Distribution, Virulence Factors, and Antifungal Susceptibility Among *Candida parapsilosis* Complex Isolates Recovered from Clinical Specimens. Mycopathologia.

[34] Pfaller M.A., Diekema D.J. (2007). Epidemiology of invasive candidiasis: A persistent public health problem. Clin. Microbiol. Rev..

[35] Kanani A., Zaini F., Kordbacheh P., Falahati M., Rezaie S., Daie R., Farahyar S., Safara M., Fateh R., Faghihloo E. (2015). Identification of Azole Resistance Markers in Clinical Isolates of *Candida tropicalis* Using cDNA-AFLP Method. J. Clin. Lab. Anal..

[36] Nabili M., Abdollahi Gohar A., Badali H., Mohammadi R., Moazeni M. (2016). Amino acid substitutions in Erg11p of azole-resistant *Candida glabrata*: Possible effective substitutions and homology modelling. J. Glob. Antimicrob. Resist..

[37] Pappas P.G., Kauffman C.A., Andes D.R., Clancy C.J., Marr K.A., Ostrosky-Zeichner L., Reboli A.C., Schuster M.G., Vazquez J.A., Walsh T.J. (2015). Clinical Practice Guideline for the Management of Candidiasis: 2016 Update by the Infectious Diseases Society of America. Clin. Infect. Dis..

[38] McCarty T.P., Pappas P.G. (2016). Invasive Candidiasis. Infect. Dis. Clin. N. Am..

[39] Pfaller M.A., Messer S.A., Rhomberg P.R., Jones R.N., Castanheira M. (2016). Activity of a long-acting echinocandin, CD101, determined using CLSI and EUCAST reference methods, against Candida and Aspergillus spp., including echinocandin- and azole-resistant isolates. J. Antimicrob. Chemother..

[40] Sanguinetti M., Posteraro B., Lass-Flörl C. (2015). Antifungal drug resistance among Candida species: Mechanisms and clinical impact. Mycoses.

[41] Mesa-Arango A.C., Trevijano-Contador N., Roman E., Sanchez-Fresneda R., Casas C., Herrero E., Arguelles J.C., Pla J., Cuenca-Estrella M., Zaragoza O. (2014). The Production of Reactive Oxygen Species Is a Universal Action Mechanism of Amphotericin B against Pathogenic Yeasts and Contributes to the Fungicidal Effect of This Drug. Antimicrob. Agents Chemother..

[42] Neofytos D., Lombardi L.R., Shields R.K., Ostrander D., Warren L., Nguyen M.H., Thompson C.B., Marr K.A. (2012). Administration of voriconazole in patients with renal dysfunction. Clin. Infect. Dis..

[43] Linder N., Klinger G., Shalit I., Levy I., Ashkenazi S., Haski G., Levit O., Sirota L. (2003). Treatment of candidaemia in premature infants: Comparison of three amphotericin B preparations. J. Antimicrob. Chemother..

[44] Ostrosky-Zeichner L., Marr K.A., Rex J.H., Cohen S.H. (2003). Amphotericin B: Time for a new “gold standard”. Clin. Infect. Dis..

[45] Safdar A., Ma J., Saliba F., Dupont B., Wingard J., Hachem R., Mattiuzzi G., Chandrasekar P., Kontoyiannis D., Rolston K. (2010). Drug-induced nephrotoxicity caused by amphotericin B lipid complex and liposomal amphotericin B: A review and meta-analysis. Medicine.

[46] Coenye T., Bjarnsholt T. (2016). The complexity of microbial biofilm research—An introduction to the 3rd Thematic Issue on Biofilms. Pathog. Dis..

[47] European Committee on Antimicrobial Susceptibility Testing Antifungal Agents Breakpoint Tables for Interpretation of MICs, Version 8.1. http://www.eucast.org/fileadmin/src/media/PDFs/EUCAST_files/AFST/Clinical_breakpoints/Antifungal_breakpoints_v_8.1_March_2017.pdf.

[48] Fielding R., Smith P., Wang L., Porter J., Guo L. (1991). Comparative pharmacokinetics of amphotericin B after administration of a novel colloidal delivery system, ABCD, and a conventional formulation to rats. Antimicrob. Agents Chemother..

[49] Carrillo-Muñoz A., Quinds G., Tur C., Ruesga M., Miranda Y., del Valle O., Cossum P., Wallace T. (1999). In Vitro antifungal activity of liposomal nystatin in comparison with nystatin, amphotericin B cholesteryl sulphate, liposomal amphotericin B, amphotericin B lipid complex, amphotericin B desoxycholate, fluconazole and itraconazole. J. Antimicrob. Chemother..

[50] Gonzalez G.M., Elizondo M., Ayala J. (2008). Trends in species distribution and susceptibility of bloodstream isolates of Candida collected in Monterrey, Mexico, to seven antifungal agents: Results of a 3-year (2004 to 2007) surveillance study. J. Clin. Microbiol..

[51] Hull C.M., Bader O., Parker J.E., Weig M., Gross U., Warrilow A.G.S., Kelly D.E., Kelly S.L. (2012). Two clinical isolates of *Candida glabrata* exhibiting reduced sensitivity to amphotericin B both harbor mutations in ERG2. Antimicrob. Agents Chemother..

[52] Hull C.M., Parker J.E., Bader O., Weig M., Gross U., Warrilow A.G.S., Kelly D.E., Kelly S.L. (2012). Facultative sterol uptake in an ergosterol-deficient clinical isolate of candida glabrata harboring a missense mutation in ERG11 and exhibiting cross-resistance to azoles and amphotericin B. Antimicrob. Agents Chemother..

[53] Pfaller M.A., Messer S.A., Hollis R.J., Jones R.N., Diekema D.J. (2002). In Vitro Activities of Ravuconazole and Voriconazole Compared with Those of Four Approved Systemic Antifungal Agents against 6, 970 Clinical Isolates of Candida spp.. Antimicrob. Agents Chemother..

[54] Groll A.H., Giri N., Petraitis V., Petraitiene R., Candelario M., Bacher J.S., Piscitelli S.C., Walsh T.J. (2000). Comparative Efficacy and Distribution of Lipid Formulations of Amphotericin B in Experimental *Candida albicans* Infection of the Central Nervous System. J. Infect. Dis..

[55] Anaissie E., Paetznick V.L., Proffitt R., Adler-Moore J., Bodey G. (1991). Comparison of the in vitro antifungal activity of free and liposome-encapsulated amphotericin B. Eur. J. Clin. Microbiol. Infect. Dis..

[56] Jessup C., Reyes G., Fothergill A., McCarthy D., Rinaldi M., Messer S., Pfaller M., Ghannoum M. (2000). A head-on comparison of the in vitro antifungal activity of conventional and lipid-based amphotericin B: A multicenter study. J. Chemother..

[57] Lass-Flörl C., Mayr A., Perkhofer S., Hinterberger G., Hausdorfer J., Speth C., Fille M. (2008). Activities of antifungal agents against yeasts and filamentous fungi: Assessment according to the methodology of the European Committee on Antimicrobial Susceptibility Testing. Antimicrob. Agents Chemother..

[58] Ito J.I., Hooshmand-Rad R. (2005). Treatment of Candida infections with amphotericin B lipid complex. Clin. Infect. Dis..

[59] Sun J., Li Z., Chu H., Guo J., Jiang G., Qi Q. (2016). *Candida albicans* Amphotericin B-Tolerant Persister Formation is Closely Related to Surface Adhesion. Mycopathologia.

[60] Boucherit Z., Seksek O., Bolard J. (2007). Dormancy of *Candida albicans* cells in the presence of the polyene antibiotic amphotericin B: Simple demonstration by flow cytometry. Med. Mycol..

[61] LaFleur M.D., Kumamoto C.A., Lewis K. (2006). *Candida albicans* biofilms produce antifungal-tolerant persister cells. Antimicrob. Agents Chemother..

[62] LaFleur M., Qingguo Q., Lewis K. (2010). Patients with long-term oral carriage harbor high-persister mutants of *Candida albicans*. Antimicrob. Agents Chemother..

[63] Al-Dhaheri R.S.R.S., Douglas L.J.J. (2008). Absence of amphotericin B-tolerant persister cells in biofilms of some Candida species. Antimicrob. Agents Chemother..

[64] Al-Dhaheri R.S., Douglas L.J. (2010). Apoptosis in Candida biofilms exposed to amphotericin B. J. Med. Microbiol..

[65] Bink A., Vandenbosch D., Coenye T., Nelis H., Cammue B.P.A., Thevissen K. (2011). Superoxide dismutases are involved in *Candida albicans* biofilm persistence against miconazole. Antimicrob. Agents Chemother..

[66] Dawson C.C., Intapa C., Jabra-Rizk M.A. (2011). “Persisters”: Survival at the cellular level. PLoS Pathog..

[67] Uppuluri P., Chaturvedi A.K., Srinivasan A., Banerjee M., Ramasubramaniam A.K., Köhler J.R., Kadosh D., Lopez-Ribot J.L. (2010). Dispersion as an important step in the *Candida albicans* biofilm developmental cycle. PLoS Pathog.

[68] Lal P., Sharma D., Pruthi P., Pruthi V. (2010). Exopolysaccharide analysis of biofilm-forming *Candida albicans*. J. Appl. Microbiol..

[69] Fonseca E., Silva S., Rodrigues C.F., Alves C.T., Azeredo J., Henriques M. (2014). Effects of fluconazole on *Candida glabrata* biofilms and its relationship with ABC transporter gene expression. Biofouling.

[70] Hawser S.P., Douglas L.J. (1995). Resistance of *Candida albicans* biofilms to antifungal agents in vitro. Antimicrob. Agents Chemother..

[71] Taff H.T., Nett J.E., Andes D.R. (2012). Comparative analysis of Candida biofilm quantitation assays. Med. Mycol..

[72] Heffner D.K., Franklin W.A. (1978). Endocarditis caused by Torulopsis glabrata. Am. J. Clin. Pathol..

[73] Tumbarello M., Fiori B., Trecarichi E.M., Posteraro P., Losito A.R., de Luca A., Sanguinetti M., Fadda G., Cauda R., Posteraro B. (2012). Risk factors and outcomes of candidemia caused by biofilm-forming isolates in a tertiary care hospital. PLoS ONE.

[74] Vila T.V.M., Rozental S. (2016). Biofilm formation as a pathogenicity factor of medically important fungi. Fungal Pathogenicity.

[75] López-Ribot J.L. (2005). *Candida albicans* biofilms: More than filamentation. Curr. Biol..

[76] Mitchell K.F., Taff H.T., Cuevas M.A., Reinicke E.L., Sanchez H., Andes D.R. (2013). Role of matrix β-1,3-glucan in antifungal resistance of non-albicans Candida biofilms. Antimicrob. Agents Chemother..

[77] Rodrigues C.F., Silva S., Azeredo J., Henrique M., Henriques M. (2015). Detection and Quantification of Fluconazole Within *Candida glabrata* Biofilms. Mycopathologia.

[78] Wingard J., White M., Anaissie E., Raffalli J., Goodman J., Arrieta A., L Amph/ABLC Collaborative Study Group (2000). A randomized, double-blind comparative trial evaluating the safety of liposomal amphotericin B versus amphotericin B lipid complex in the empirical treatment of febrile neutropenia. Clin. Infect. Dis..

[79] Astellas Pharma US, Inc. (2012). AmBisome (Amphotericin B) Liposome for Injection.

[80] Vandeputte P., Tronchin G., Larcher G., Ernoult E., Bergès T., Chabasse D., Bouchara J.-P. (2008). A nonsense mutation in the ERG6 gene leads to reduced susceptibility to polyenes in a clinical isolate of *Candida glabrata*. Antimicrob. Agents Chemother..

[81] Vandeputte P., Tronchin G., Bergès T., Hennequin C., Chabasse D., Bouchara J.-P. (2007). Reduced susceptibility to polyenes associated with a missense mutation in the ERG6 gene in a clinical isolate of *Candida glabrata* with pseudohyphal growth. Antimicrob. Agents Chemother..

[82] Ferrari S., Sanguinetti M., De Bernardis F., Torelli R., Posteraro B., Vandeputte P., Sanglard D. (2011). Loss of mitochondrial functions associated with azole resistance in *Candida glabrata* results in enhanced virulence in mice. Antimicrob. Agents Chemother..

[83] Choi H.W., Shin J.H., Jung S.I., Park K.H., Cho D., Kee S.J., Shin M.G., Suh S.P., Ryang D.W. (2007). Species-specific differences in the susceptibilities of biofilms formed by Candida bloodstream isolates to echinocandin antifungals. Antimicrob. Agents Chemother..

[84] Kuhn D.M., George T., Chandra J., Mukherjee P.K., Ghannoum M.A. (2002). Antifungal Susceptibility of Candida Biofilms: Unique Efficacy of Amphotericin B Lipid Formulations and Echinocandins. Antimicrob. Agents Chemother..

[85] Liao Z., ZhangGuan X., Zhu Z., Yao X., Yang Y., Jiang Y., Cao Y. (2014). Enhancement of the antibiofilm activity of amphotericin B by polyamine biosynthesis inhibitors. Int. J. Antimicrob. Agents.

[86] Luiz R.L.F., Vila T.V.M., de Mello J.C.P., Nakamura C.V., Rozental S., Ishida K. (2015). Proanthocyanidins polymeric tannin from Stryphnodendron adstringens are active against *Candida albicans* biofilms. BMC Complement. Altern. Med..

[87] Kvasnickova E., Matatkova O., Cejkova A., Masak J. (2015). Evaluation of baicalein, chitosan and usnic acid effect on *Candida parapsilosis* and *Candida krusei* biofilm using a Cellavista device. J. Microbiol. Methods.

[88] Mahl C.D., Behling C.S., Hackenhaar F.S., de Carvalho E Silva M.N., Putti J., Salomon T.B., Alves S.H., Fuentefria A., Benfato M.S. (2015). Induction of ROS generation by fluconazole in *Candida glabrata*: Activation of antioxidant enzymes and oxidative DNA damage. Diagn. Microbiol. Infect. Dis..

[89] Marcos-Zambrano L., Escribano P., Bouza E., Guinea J. (2016). Comparison of the antifungal activity of micafungin and amphotericin B against *Candida tropicalis* biofilms. J. Antimicrob. Chemother..

[90] Zahran K.M., Agban M.N., Ahmed S.H., Hassan E.A., Sabet M.A. (2015). Patterns of candida biofilm on intrauterine devices. J. Med. Microbiol..

[91] Shanmughapriya S., Sornakumari H., Lency A., Kavitha S., Natarajaseenivasan K. (2014). Synergistic effect of amphotericin B and tyrosol on biofilm formed by *Candida krusei* and *Candida tropicalis* from intrauterine device users. Med. Mycol..

[92] Maiolo E.M., Tafin U.F., Borens O., Trampuz A. (2014). Activities of fluconazole, caspofungin, anidulafungin, and amphotericin B on planktonic and biofilm candida species determined by microcalorimetry. Antimicrob. Agents Chemother..

[93] Aslan H., Gulmez D. (2016). Investigation of the correlation between biofilm forming ability of urinary Candida isolates with the use of urinary catheters and change of antifungal susceptibility in the presence of biofilm. Mikrobiyol. Buleni.

[94] Seneviratne C.J., Wang Y., Jin L., Abiko Y., Samaranayake L.P., Jayampath Seneviratne C., Wang Y., Jin L., Abiko Y., Samaranayake L.P. (2010). Proteomics of drug resistance in candida glabrata biofilms. Proteomics.

[95] Rodrigues C.F., Silva S., Azeredo J., Henriques M. (2016). *Candida glabrata*’s recurrent infections: Biofilm formation during Amphotericin B treatment. Lett. Appl. Microbiol..

[96] Ibrahim N.H., Melake N.A., Somily A.M., Zakaria A.S., Baddour M.M., Mahmoud A.Z., Arthington-Skaggs B.A., Jradi H., Desai T., Morrison C.J. (2015). The effect of antifungal combination on transcripts of a subset of drug-resistance genes in clinical isolates of Candida species induced biofilms. Antimicrob. Agents Chemother..

[97] Miyazaki T., Kohno S. (2014). ER stress response mechanisms in the pathogenic yeast *Candida glabrata* and their roles in virulence. Virulence.

[98] Prasanna K.D., Suci P.A., Miller R.L., Nelson R.D., Tyler B.J. (2006). A Small Subpopulation of Blastospores in *Candida albicans* Biofilms Exhibit Resistance to Amphotericin B Associated with Differential Regulation of Ergosterol and B-1,6-Glucan Pathway Genes. Antimicrob. Agents Chemother..

[99] Arendrup M.C., Arikan S., Barchiesi F., Bille J., Dannaoui E., Denning D.W., Donnelly J.P., Fegeler W., Moore C., Richardson M. (2008). EUCAST Technical Note on the method for the determination of broth dilution minimum inhibitory concentrations of antifungal agents for conidia—Forming moulds. Clin. Microbiol. Infect..

[100] Alastruey-Izquierdo A., Cuenca-Estrella M. (2012). EUCAST and CLSI: How to assess in vitro susceptibility and clinical resistance. Curr. Fungal Infect. Rep..

[101] Rodrigues C.F., Gonçalves B., Rodrigues M.E., Silva S., Azeredo J., Henriques M. (2017). The Effectiveness of Voriconazole in Therapy of *Candida glabrata*’s Biofilms Oral Infections and Its Influence on the Matrix Composition and Gene Expression. Mycopathologia.

[102] Rodrigues C., Henriques M. (2017). Oral mucositis caused by *Candida glabrata* biofilms: Failure of the concomitant use of fluconazole and ascorbic acid. Ther. Adv. Infect. Dis..

